# Advantageous Direct Quantification of Viable Closely Related Probiotics in *Petit-Suisse* Cheeses under *In Vitro* Gastrointestinal Conditions by Propidium Monoazide - qPCR

**DOI:** 10.1371/journal.pone.0082102

**Published:** 2013-12-17

**Authors:** Martha Lissete Morales Villarreal, Marina Padilha, Antonio Diogo Silva Vieira, Bernadette Dora Gombossy de Melo Franco, Rafael Chacon Ruiz Martinez, Susana Marta Isay Saad

**Affiliations:** 1 Department of Biochemical and Pharmaceutical Technology, Faculty of Pharmaceutical Sciences, University of São Paulo, São Paulo, SP, Brazil; 2 Department of Food and Experimental Nutrition, Faculty of Pharmaceutical Sciences, University of São Paulo, São Paulo, SP, Brazil; Beijing Institute of Microbiology and Epidemiology, China

## Abstract

Species-specific Quantitative Real Time PCR (qPCR) alone and combined with the use of propidium monoazide (PMA) were used along with the plate count method to evaluate the survival of the probiotic strains *Lactobacillus acidophilus* La-5 and *Bifidobacterium animalis* subsp. *lactis* Bb-12, and the bacteriocinogenic and potentially probiotic strain *Lactobacillus sakei* subsp. *sakei* 2a in synbiotic (F1) and probiotic (F2) *petit-suisse* cheeses exposed throughout shelf-life to *in vitro* simulated gastrointestinal tract conditions. The three strains studied showed a reduction in their viability after the 6 h assay. Bb-12 displayed the highest survival capacity, above 72.6 and 74.6% of the initial populations, respectively, by plate count and PMA-qPCR, maintaining population levels in the range or above 6 log CFU/g. The prebiotic mix of inulin and FOS did not offer any additional protection for the strains against the simulated gastrointestinal environment. The microorganisms' populations were comparable among the three methods at the initial time of the assay, confirming the presence of mainly viable and culturable cells. However, with the intensification of the stress induced throughout the various stages of the *in vitro* test, the differences among the methods increased. The qPCR was not a reliable enumeration method for the quantification of intact bacterial populations, mixed with large numbers of injured and dead bacteria, as confirmed by the scanning electron microscopy results. Furthermore, bacteria plate counts were much lower (*P*<0.05) than with the PMA-qPCR method, suggesting the accumulation of stressed or dead microorganisms unable to form colonies. The use of PMA overcame the qPCR inability to differentiate between dead and alive cells. The combination of PMA and species-specific qPCR in this study allowed a quick and unequivocal way of enumeration of viable closely related species incorporated into probiotic and synbiotic *petit-suisse* cheeses and under stress conditions.

## Introduction

Lactic acid bacteria (LAB) comprise a group of several genera of microorganisms, which display lactic acid as their major end product of fermentation [1]. LAB are indigenous members of the intestinal microbiota of animals and are also associated with plants, and fermented food, including meat and dairy products [2]. These microorganisms produce different antimicrobial peptides, known as bacteriocins, which are useful to improve the safety and biopreservation of foods [3].

Probiotic microorganisms are defined as ‘live microorganisms, which when administered in adequate amounts confer health benefits to the host’ [4]. The consumption of probiotics are related to diverse human health benefits, including the homeostasis of intestinal microbiota, anticarcinogenesis, hypocholesterolemic effect, reduction in the risk of diarrhea caused by bacteria and virus, alleviation of lactose malabsorption, and allergy [5], [6]. Species of *Lactobacillus* and *Bifidobacterium* are most commonly used as probiotics. Among them, *Bifidobacterium animalis* subsp. *lactis* Bb-12 and *Lactobacillus acidophilus* La-5 strains have been widely studied with several health benefits associated to their consumption [7], [8]. Bacteriocin-producing *Lactobacillus* species might play a dual role by acting as agents for food preservation and safety as well as presenting a potential to confer health benefits [9]. The *Lactobacillus sakei* subsp. *sakei* 2a strain, isolated from Brazilian sausage, was found to produce a bacteriocin which is active against *Listeria monocytogenes* and *Staphylococcus aureus* [10]. On the other hand, prebiotics are fermentable ingredients that allow specific changes in the composition and/or activity of the gastrointestinal microbiota leading to benefits in host health and well-being [11].

Functional foods, including pro- and prebiotics, beyond basic nutritional functions, show physiological benefits and can help to maintain the gut health [12]. Dairy matrices are traditionally used as vehicles for probiotics delivery. Among them, cheeses are of special importance, since, in comparison to yogurts and fermented milks, they present higher pH, less acidity, higher buffering capacity, higher lipid content and nutrients availability, and lower oxygen levels, resulting in a higher degree of protection to the microorganisms during their passage throughout the harsh conditions found in the gastrointestinal tract (GIT) [13]. Due to these characteristics, the successful use of cheeses supplemented with prebiotics to deliver probiotic cultures has been reported [14], [15].

The probiotic potential of a microorganism might be assessed using *in vitro* models that mimic the physical–chemical conditions found in the human GIT tract, including survival at low pH (stomach environment) and the presence of bile salts and digestive enzymes (simulating the duodenum characteristics) [16]. Interestingly, according to van Bokhorst-van de Veen et al. [17], there is a considerable variation in the GIT survival when different strains of a same microorganism are evaluated.

Although there is not a standardized amount of live microorganisms that must reach the colon, it is believed that this number correlates with the health benefits intended [18] and, therefore, it is important to quantify their survival under the different steps of the gastric and enteric conditions. For the detection and quantification of microbial populations, plating on selective media has been traditionally used. However, culture-dependent techniques have been questioned due to their inability to differentiate viable cells from non-culturable ones. The depletion of nutrients, low pH, and the enzymatic activity are conditions able to promote injury and may alter the cell metabolic activity, limiting the ability of the microorganisms to form colonies, and this fact may impair the identification and underestimate their quantification, mainly in survival assays [19].

Molecular methods have now overcome, at a great extent, the problem of lack of sensibility and specificity of conventional culture-dependent techniques, and the Quantitative Real-Time PCR (qPCR) is, currently, the most used method for direct quantification of microorganisms in complex samples [20]. However, its main problem is the inability to differentiate live and dead microorganisms cells, since DNA can be amplified even if the cells are dead [21]. In order to solve this pitfall, the use of DNA intercalating agents that inhibit its amplification has been studied, among which are the ethidium monoazide (EMA) and propidium monoazide (PMA) [22]. The main criterion for distinguishing between viable and irreversibly damaged cells is the membrane activity, and since these intercalating agents can only penetrate cells with damaged membranes [21], [23], their use in combination with qPCR method allows a quantitative and differential detection of viable *versus* non-viable microorganisms in a given microbial community. Furthermore, PMA is activated upon light exposure and was shown to exert no toxic effect on viable cells [24].

The aim of this study was to evaluate the survival of the probiotic strains *L. acidophilus* La-5 and *B. animalis* subsp. *lactis* Bb-12 and of the bacteriocinogenic and potentially probiotic strain *L. sakei* subsp. *sakei* 2a in synbiotic and probiotic *petit-suisse* cheeses throughout their shelf-lives challenged by an *in vitro* assay simulating gastrointestinal conditions. The suitability of three different enumeration methods for a reliable determination of the bacterial population under the stressful conditions tested was also evaluated, including plate count, species-specific qPCR alone and combined with the use of the PMA, for which the results obtained were compared and fully discussed.

## Materials and Methods

### Bacterial Strains and Culture Conditions

Commercial DVS culture, ABT-4, containing the probiotic strains *L. acidophilus* La-5 and *B. animalis* subsp. *lactis* Bb-12 and the starter culture *Streptococcus thermophilus* was obtained from Christian Hansen laboratories (Hoersholm, Denmark). ABT-4 culture was added directly to commercial pasteurized skimmed milk (Salute, Descalvado, Brazil) at 1% (v/v) in order to achieve ca. 8.0 log CFU/g of each strain in the final product. The strain *L. sakei* subsp. *sakei* 2a, belonging to the collection of the Laboratory of Food Microbiology (Faculty of Pharmaceutical Sciences -University of São Paulo; FCF-USP), was cultured overnight at 30°C in de Man, Rogosa and Sharpe (MRS) (Oxoid, Basingstoke, UK) broth and harvested by centrifugation at 9000 *g* for 5 min at 4°C. *L. sakei* 2a was added to the product in order to reach a final concentration of ca. 8.0 log CFU/g.

### Probiotic *Petit-Suisse C*heeses Production

Two pilot-scale *petit*-*suisse* cheeses formulations, denoted F1 (synbiotic) and F2 (probiotic), were manufactured. Two batches of both F1 and F2 were produced, independently, at different days, as described by Cardarelli et al. [25]. Briefly, cultures were added to pre-heated (37°C) commercial pasteurized skimmed milk for the manufacture of the cheese-base (quark cheese); next, it was incubated until pH 6.3–6.5 (approximately 1 h) before the addition of rennet Ha-la (Christian Hansen, Valinhos, Brazil, 0.03 g/L). After curd formation, it was cut and drained in sterilized cotton cheesecloth. The quark cheese was allowed to drain at 15°C for 15 h before homogenization and mixture of the remaining ingredients (Table 1). Portions of 50 g of the *petit-suisse* cheeses were packaged in polypropylene plastic pots for food products (68 mm diameter, 32 mm high, 55 mL total volume, Tries Aditivos Plásticos, São Paulo, Brazil), sealed with an aluminum cover, stored at 4±1°C, and sampled on days 1, 14, and 28.

**Table 1:** Ingredients and respective proportions used in the preparation of the *petit-suisse* cheeses.

Ingredient	Proportion added (g/100 g)	
	Synbiotic cheese (F1)	Probiotic cheese (F2)
Quark cheese	57.50	61.970
Sterilized milk cream (25% fat, Nestlé, Araçatuba, Brazil)	12.00	13.720
Pasteurized strawberry whole pulp (Ice-Fruit-Maisa, Mossoró, Brazil)	10.00	11.500
Sucrose (União, Limeira, Brazil)	9.00	11.000
Guar gum (Grindsted® Guar 250 Danisco, Cotia, Brazil)	0.20	0.300
Xanthan gum (Grindsted® Xanthan 80, Danisco)	0.15	0.187
Carrageenan gum (Grindsted® Carrageenan CY 500, Danisco)	0.15	0.187
Natural coloring and flavor agent (7134, Germinal, Cabreúva, Brazil)	1.00	1.120
Inulin (Beneo GR, Orafti, Oreye, Belgium)	7.50	-
FOS (Fructooligosacchrides, Beneo™ P95, Orafti)	2.50	-

### Probiotic and Synbiotic *Petit-Suisse* Cheeses and Gastrointestinal Tract Transit Simulation

The *in vitro* assay simulating the gastrointestinal conditions (gastric, enteric I, and enteric II phases) was carried out according the protocol of Liserre et al. [26] modified by Buriti et al. [27]. Some adjustments were made and are described as follows. Firstly, at each sampling day, 25 g of three different samples belonging to each batch from the same formulation (F1 and F2, n = 6) of *petit-suisse* cheeses were homogenized with 225 mL of sterilized sodium chloride (NaCl) solution (0.9%, w/v), using a BagMixer® 400P (Interscience, St. Nom, France) during 3 min. Aliquots of all samples were transferred in triplicates to three sterilized flasks (9 flasks in total) and incubated in a water bath at 37°C with orbital agitation of 150 rpm (Metabolic Water Bath Dubnoff MA-095, Marconi, Piracicaba, Brazil). The whole process lasted a total of six hours during which the pH of the samples was adjusted and various gastrointestinal enzymes were added according to the gastrointestinal phase simulated (Table 2). Aliquots were collected at time-points zero and after 2 h (gastric phase), 4 h (enteric phase I), and 6 h (enteric phase II) for analysis.

**Table 2:** Description of phases employed in the *in vitro* simulation of gastrointestinal transit.

Phase	Description
1. Gastric	pH adjustment to pH 2.3–2.6 with 1N HCl (Merck).
	Addition of pepsin^1^ and lipase^1^ to homogenate, 2 h incubation
2. Enteric I	pH adjustment to pH 5.4-5.7 with alkaline solution^2^
	Addition of pancreatin^1^ and bile^1^ to homogenate, 2 h incubation
3. Enteric II	pH adjustment to pH 6.8-7.2 with alkaline solution^2^
	Addition of pancreatin and bile to homogenate, 2 h incubation

**Footnote:**

1 Enzymes: 3.0 g/L pepsine; 0.9 mg/L lipase; 10 g/L bile; 1.0 g/L pancreatin (all from Sigma – Aldrich, St. Louis, USA).

2 Alkaline solution: 150 mL of 1N NaOH (Synth, Diadema, Brazil) and 14 g of PO_4_H_2_Na. 2H_2_O (Synth) (distilled water up to 1L).

To better assess the survival rate (R) of the strains under stress, the percentage of bacterial recovery was calculated as follows:
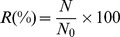
where N is the log of CFU of bacteria assessed after a particular *in vitro* phase and N_0_ is the initial log of CFU of bacteria determined at the same phase.

### Assay Samples Analysis

#### Enumeration of Bacteria by the Plate Count Method

Standard pour-plate method was used for viable microbial enumeration. The culture media included: MRS agar supplemented with both sodium propionate (3.0 g/L) (Sigma-Aldrich, St. Louis, MO, USA) and lithium chloride (2.0 g/L) (Merck, Darmstadt, Germany) (MRS-LP) for quantification of *B. animalis* Bb-12 as described by Vinderola and Reinheimer [28]; MRS agar modified by the replacement of glucose by maltose (Difco, Le Pont de Claix, France) according to the International Dairy Federation [29] for the enumeration of La-5 and MRS agar (Oxoid) for quantification of *L. sakei* 2a. Bb-12 was incubated under anaerobic conditions using the Anaerobic System Anaerogen (Oxoid) at 37°C for 72 h, whereas La-5 and *L. sakei* 2a were incubated aerobically at 37°C and 30°C, respectively, for 48 h.

#### Evaluation of Bacterial Survival by Quantitative PCR

In order to determine the survival of the studied microorganisms throughout the *in vitro* simulated gastrointestinal tract conditions, total DNA isolation from the *in vitro* samples proceeded as follows. Aliquots of 3 mL, obtained as described above, were homogenized with 27 mL of trisodium citrate dehydrate solution (2%, w/v) and incubated at 45°C for 30 min. The suspensions obtained were centrifuged at 9000 *g* for 10 min at 4°C. The supernatant was discarded and the pellet washed, resuspended in 500 µL of Tris EDTA (10 mM Tris-HCl, 1 mM EDTA, [pH 8]) buffer and transferred to a 2 mL screw-cap tube containing 0.3 g of 0.1 mm zirconia beads (Biospec, Bartlesville, OK, USA) plus 150 µL of buffer-saturated phenol (Invitrogen, Carlsbad, CA, USA). The samples were lyzed using a FastPrep®-24 bead-beater (MP Biomedical, Solon, OH, USA) and the initial procedure was repeated three consecutive times at a speed of 5.0 m/s for 30 s, interspaced by breaks of 1 min, during which samples were kept on ice. Total DNA was isolated by successive phenol-chloroform∶isoamyl alcohol extractions, until a clear interface was obtained, and used for purification through ethanol precipitation. DNA was collected by centrifugation and resuspended in 50 µL of TE buffer. DNA quality and concentrations were determined using a Nanodrop ND-1000 (Thermo Scientific, Waltham, USA).

The PMA (phenanthridium, 3-amino-8-azido-5-[3-(diethylmethylammonio)propyl]-6- phenyl dichloride) treatment was carried out as described by Nocker et al. [21], with slight modifications. The samples were prepared as described in item 2.3.2.1 up to the step in which the pellet was resuspended in 500 µL of buffer TE (pH 8.0). Following, the suspension was transferred to 1.5 mL light-transparent microcentrifuge tubes containing 1.25 µL of a 20 mM stock solution of PMA (Biotium, Inc., Hayward, CA, USA) (previously prepared in 20% dimethyl sulfoxide) to reach a final concentration of 50 µM and finally mixed. After a dark incubation during 5 min, the samples were light-exposed for 15 min by use of a 650-W halogen light device (DWE, 650 W, 120 V, GE Lighting, East Cleveland, Ohio, USA). The tubes containing the samples were laid horizontally on ice, to avoid excessive heating, and placed about 20 cm away from the light source. After photo-induced cross-linking, cells were pelleted at 10000 *g*, for 10 min at 4°C. The supernatant obtained was discarded and the pellet washed with PBS buffer in order to remove the inactivated PMA. The cells were collected by centrifugation and resuspended in 500 µL of buffer TE (pH 8.0) prior to DNA isolation procedure, as described above.

For viable microbial quantification, all quantitative PCR (qPCR) reactions were performed by using the ABI-PRISM 7500 sequencing detection system (Applied Biosystems, Bridgewater, NJ, USA). The final volume for all reactions was 25 µL, among them, 20 µL consisted of the PCR Master Mix and 5 µl of DNA extracted with and without use of PMA treatment.

For the La-5 assay, 1× Power SYBR Green PCR Master Mix (Applied Biosystems) and 400 nM of each primer was used. The primers and the amplification program used were the same ones previously described by Tabasco et al. [30]. The melting curve analysis was performed after amplification reaction to distinguish the target from the non-targeted PCR products. In order to quantify Bb-12 populations, the PCR reaction mix contained 1× TaqMan Master Mix (Applied Biosystems), 300 nM of BAL-23S-F forward and BAL-23S-R reverse primers, and 250 nM of BAL-23S-P probe, as described by Taipale et al. [31]. Finally, for the enumeration of *L. sakei* 2a, the PCR reaction mix consisted of 1× TaqMan Master Mix (Applied Biosystems), 900 nM of each SakF forward and SakR reverse primers, and 200 nM of SakS minor groove binder (MGB) probe [32]. The amplification profile program used for enumeration of Bb-12 and *L. sakei* was: 50°C for 2 min, 95°C for 10 min, and 40 cycles of 95°C for 15 s and 60°C for 30 s.

For the determination of the sensitivity and the amplification efficiency of the qPCR reactions, a standard curve was constructed for each microorganism. The standard curves for *L. sakei* 2a and Bb-12 consisted of purified genomic DNA isolated from pure cultures serially diluted with sterilized nuclease-free water for obtaining a range of DNA concentrations equivalent to approximately 5×10^7^ to 5 genome copies of each microorganism per amplification reaction mixture. The number of Bb-12 and *L. sakei* 2a genome equivalents was estimated by considering the previously known genome size of Bb-12 [33] and *L. sakei* 23K [34] as reference microorganisms. Consequently, the equivalent of one genome of Bb-12 and *L. sakei* 2a weighs, approximately 2.13 and 2 fg, respectively. The quantification of La-5 was done against a 10-fold dilution series of 16S rRNA gene fragment equivalent to 5×10^7^ to 5 copy number of the gene. The approximately 1500-bp 16S rRNA gene fragment was obtained by PCR amplification of genomic DNA from pure culture of La-5 using universal primers 27F and 1492R and conditions described by Felske and Weller [35]. The amplified fragment was checked by using agarose gel electrophoresis and purified with the QIAquik PCR Purification kit (QIAGEN, Hilden, Germany), according to the manufacturer's instructions. Considering the genome of *L. acidophilus* NCFM as reference strain, the copy number of 16S rRNA gene in La-5 genome was estimated at 4 [36].

The coefficients of efficiency of the PCR amplifications, calculated as 10^(−1/slope)^ -1, were: 95.6%; 100%, and 99%, respectively, for Bb-12, *L. sakei* 2a, and La-5. The correlation coefficients (R^2^) of the standard curves obtained were between 0.995 and 0.998 for the initial copy numbers of standards within the range covered per reaction. Non-template controls (NTC) samples were included in all PCR runs and tested negative. The experiment was repeated two independent times, with triplicates within each assay.

#### Scanning Electron Microscopy (SEM)

Samples obtained as described above were centrifuged at 5000 *g* for 10 min, and the supernatant was discarded. The cell pellets were resuspended in NaCl solution (0.9%, w/v) at a final concentration of ca. 5 log CFU/ mL. Aliquots of 1 mL of the cell suspensions were placed onto 0.2 µm-pore size membrane filter (Isopore membrane filters, Millipore, Billerica, MA, USA) and fixed for 2 h in 2% (w/v) glutaraldehyde solution. Subsequently, the membranes were washed three times with Milli-Q water (Millipore), dehydrated sequentially with 25, 50, 75, 90, and 95% ethanol solutions and finally with 100% ethanol (three times), and critical- point dried by the CO_2_ method. The dried membranes were placed on aluminum stubs, sputter coated with gold and analyzed by using a field emission scanning electron microscope (JEOL JSM-7401F; JEOL, Tokyo, Japan) at 2.5 kV at the facilities of the Chemistry Institute (IQ), at the University of São Paulo, Brazil.

### Statistical Analysis

The C_T_ values obtained by the qPCR method and used to quantify the viable cells (CFU/g) were automatically generated by the ABI-PRISM 7500 software. One-way analysis of variance (ANOVA) with post-hoc Tukey test was used to compare the results obtained by qPCR with and without the use of PMA treatment, and by the plate counts from the three phases of the *in vitro* assay, at every sampled day. The XLSTAT 2013 (Addinsoft, SARL, New York, USA) and MINITAB 14 (Minitab Inc., State College, USA) software were used to determine the statistical significance of the results obtained.

## Results

### Quantification of Viable Microorganisms in the *Petit-Suisse C*heeses Stored at 4±1°C, Throughout 28 Days Under Digestive Stress Conditions

The quantification of viable cells was evaluated before and during the different stages of the simulated digestive stress and throughout the storage time evaluated (28 days) of the *petit-suisse* cheeses using three different methodologies (culture, qPCR, and PMA-qPCR). In this study, the specificity of the three sets of primers used in the molecular methods were checked and no cross reactions were observed in the *petit-suisse* cheese formulations, with any of the non-target microorganism tested. On the contrary, four different selective media were employed and up to 72 h were required for enumeration of microorganisms. Bb-12 grew slowly and formed small colonies in MRS-LP agar, whereas the modified MRS agar, employed for La-5 enumeration, allowed the growth of few lenticular colonies of *L. sakei* 2a, different from the typical star shape colonies of La-5.

**Figure 1:**
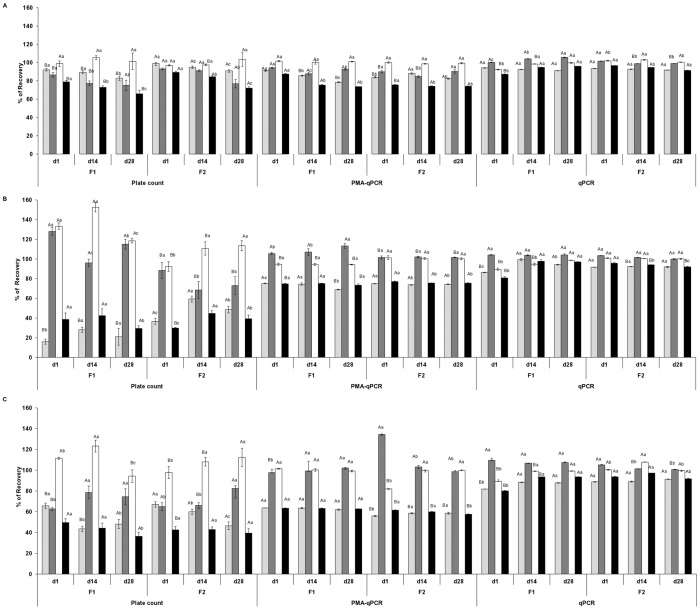
Bacterial recovery rates throughout the *in vitro* assay simulating the gastrointestinal tract conditions. Recovery (%) expressed as the ratio of live cells of *B. animalis* Bb-12 (**A**), *L. acidophilus* La-5 (**B**), and *L. sakei* 2a (**C**) before and after every step throughout the *in vitro* assay simulating the gastrointestinal tract conditions, for each storage time and both formulations (F1 - synbiotic; F2 - prebiotic). (light gray columns) after 2 h of gastric phase (pH 2.3–2.6 in the presence of pepsin and lipase); (dark gray columns) after 2 h of enteric phase I (pH 5.4–5.7 in the presence of pancreatin and bile); (white columns) after 2 h of enteric phase II (pH 6.8–7.2 in the presence of pancreatin and bile) and (black columns) total recovery after 6 hours relative to the initial inocula of cheeses. **Footnote:** ^A,B,C^ Different superscript capital letters denote significant differences between formulations for the same storage period and assay phase (*P*<0.05). ^a,b,c^ Different superscript lowercase letters denote significant differences between storage days for the same phase (*P*<0.05).

As can be seen in Tables 3 and 4, the viability results of the microbial species in the cheeses were influenced by the method employed and the values obtained were statistically different (*P*<0.05). Furthermore, higher numbers of viable cells were always detected by the qPCR method, in comparison with the values obtained using the plate counts and the PMA-qPCR techniques, regardless the strain or the *in vitro* phase analyzed. The strains in the cheese matrixes showed different patterns of survival during each phase of simulated digestive stress. Although the sensitivity to low pH and digestive enzymes was observed to be strain-specific, the bacterial survival ratios obtained by plate counting and PMA-qPCR methods were lower than those obtained by qPCR (*P*<0.05) (Fig. 1). The survival rates obtained for La-5, Bb-12, and *L. sakei* 2a were always above 90%, when the qPCR method was used, showing its inability to distinguish between live and dead cells, particularly with higher background of dead bacteria, as observed at the end of the 6^th^ h assay. Since the accurate enumeration of viable cells was the main objective of this study, the qPCR method was withdrawn from the subsequent discussion performed in here.

**Table 3:** Populations of *B. animalis* Bb-12, *L. acidophilus* La-5, and *L. sakei* 2a assessed in the synbiotic *petit-suisse* cheese (F1) throughout storage (1, 14, and 28 days) at 4°C and during the *in vitro* assay simulating the gastrointestinal conditions [time-point zero, and after 2 h (gastric phase), 4 h (enteric phase I), and 6 h (enteric phase II)].

			Method of analysis		
Species	Storage period (days)	*In vitro* phase	Plate Count	qPCR	PMA-qPCR
*B. animalis* Bb-12	1	0	8.70±0.06^C^ ^a^	9.84±0.02^A^	9.10±0.01^B^ ^a^
		gastric	8.03±0.09^C^ ^a^	9.29±0.03^A^	8.31±0.04^B^ ^a^
		enteric I	6.95±0.16^C^ ^a^	9.31±0.01^A^	7.83±0.03^B^ ^a^
		enteric II	6.89±0.13^C^ ^a^	8.60±0.02^A^	7.95±0.06^B^ ^a^
	14	0	8.39±0.06^C^ ^b^	9.81±0.02^A^	9.03±0.05^B^ ^b^
		gastric	7.48±0.13^C^ ^b^	9.07±0.02^A^	7.74±0.02^B^ ^b^
		enteric I	5.81±0.16^C^ ^b^	9.44±0.01^A^	6.79±0.12^B^ ^b^
		enteric II	6.14±0.09^C^ ^b^	9.30±0.02^A^	6.81±0.04^B^ ^b^
	28	0	8.27±0.05^C^ ^b^	9.99±0.02^A^	9.16±0.03^B^ ^a^
		gastric	6.94±0.22^B^ ^c^	9.11±0.01^A^	7.19±0.08^B^ ^c^
		enteric I	5.05±0.41^C^ ^c^	9.63±0.02^A^	6.68±0.02^B^ ^b^
		enteric II	5.52±0.32^C^ ^c^	9.59±0.02^A^	6.76±0.01^B^ ^b^
*L. acidophilus* La-5	1	0	8.40±0.07^B^ ^a^	8.75±0.04^A^	7.97±0.01^C^ ^a^
		gastric	5.47±0.19^B^ ^a^	7.16±0.02^A^	5.08±0.01^C^ ^a^
		enteric I	3.96±0.66^C^ ^a^	7.84±0.01^A^	4.99±0.04^B^ ^a^
		enteric II	4.11±0.28^C^ ^a^	7.00±0.01^A^	5.06±0.02^B^ ^a^
	14	0	8.15±0.04^B^ ^b^	8.54±0.04^A^	7.81±0.01^C^ ^b^
		gastric	3.44±0.30^C^ ^b^	7.55±0.03^A^	4.96±0.05^B^ ^ab^
		enteric I	3.00±0.35^C^ ^b^	8.06±0.01^A^	4.93±0.03^B^ ^a^
		enteric II	3.67±0.38^C^ ^b^	7.98±0.03^A^	4.94±0.02^B^ ^a^
	28	0	8.09±0.05^B^ ^b^	8.75±0.03^A^	7.79±0.04^C^ ^b^
		gastric	3.98±0.39^C^ ^b^	7.68±0.01^A^	4.82±0.07^B^ ^b^
		enteric I	2.91±0.29^C^ ^b^	8.24±0.01^A^	4.93±0.02^B^ ^a^
		enteric II	2.98±0.28^C^ ^c^	8.18±0.02^A^	4.89±0.01^B^ ^a^
*L. sakei* 2a	1	0	7.78±0.07^C^ ^a^	9.32±0.03^A^	8.00±0.03^B^ ^a^
		gastric	1.25±0.19^C^ ^c^	8.05±0.03^A^	6.01±0.02^B^ ^a^
		enteric I	1.97±0.32^C^ ^a^	8.39±0.02^A^	6.34±0.05^B^ ^a^
		enteric II	2.96±0.46^C^ ^a^	7.52±0.08^A^	6.01±0.03^B^ ^a^
	14	0	7.69±0.12^C^ ^a^	8.69±0.03^A^	7.97±0.03^B^ ^a^
		gastric	2.25±0.29^C^ ^a^	8.64±0.06^A^	5.93±0.10^B^ ^a^
		enteric I	2.05±0.11^C^ ^a^	8.97±0.01^A^	6.35±0.09^B^ ^a^
		enteric II	3.30±0.46^C^ ^a^	8.50±0.11^A^	5.99±0.03^B^ ^a^
	28	0	7.47±0.10^C^ ^b^	9.00±0.04^A^	7.97±0.02^B^ ^a^
		gastric	1.70±0.66^C^ ^b^	8.49±0.02^A^	5.48±0.06^B^ ^b^
		enteric I	2.19±0.18^C^ ^a^	8.84±0.01^A^	6.21±0.10^B^ ^a^
		enteric II	2.63±0.42^C^ ^a^	8.73±0.02^A^	5.86±0.12^B^ ^a^

Populations of *B. animalis* Bb-12, *L. acidophilus* La-5, and *L. sakei* 2a assessed in the synbiotic *petit-suisse* cheese (F1) throughout storage (1, 14, and 28 days) at 4°C and during the *in vitro* assay simulating the gastrointestinal conditions [time-point zero, and after 2 h (gastric phase), 4 h (enteric phase I), and 6 h (enteric phase II)]. The samples were analyzed using three methods: plate count, Real-time PCR (qPCR) and Real-time PCR combined with propidium monoazide (PMA-qPCR).

**Footnote:**

Values are expressed as mean log CFU/g ± standard deviation (SD) obtained by plate count method and as log CFU equivalents/g as calculated from Ct values for qPCR and PMA-qPCR;

A,B,C Different superscript capital letters in a row denote significant differences between methods (*P*<0.05).

a,b,c Different superscript lowercase letters in the same column for each phase denote significant differences between storage days (*P*<0.05).

**Table 4:** Populations of *B. animalis* Bb-12, *L. acidophilus* La-5, and *L. sakei* 2a assessed in the probiotic *petit-suisse* cheese (F2) throughout storage (1, 14, and 28 days) at 4°C and during the *in vitro* assay simulating the gastrointestinal conditions [time-point zero, and after 2 h (gastric phase), 4 h (enteric phase I), and 6 h (enteric phase II)].

			Method of analysis		
Species	Storage period (days)	*In vitro* phase	Plate Count	qPCR	PMA-qPCR
*B. animalis* Bb-12	1	0	8.82±0.08^C^ ^a^	9.94±0.02^A^	9.00±0.05^B^ ^a^
		gastric	8.69±0.07^B^ ^a^	9.28±0.03^A^	7.55±0.05^C^ ^b^
		enteric I	8.09±0.06^B^ ^a^	9.44±0.01^A^	6.79±0.05^B^ ^a^
		enteric II	7.87±0.05^B^ ^a^	9.62±0.03^A^	6.80±0.04^B^ ^a^
	14	0	8.83±0.06^C^ ^a^	9.95±0.04^A^	8.98±0.03^B^ ^a^
		gastric	8.35±0.14^B^ ^a^	9.25±0.02^A^	7.92±0.05^C^ ^a^
		enteric I	7.63±0.13^B^ ^b^	9.14±0.02^A^	6.72±0.08^B^ ^a^
		enteric II	7.46±0.08^B^ ^b^	9.41±0.03^A^	6.67±0.05^B^ ^b^
	28	0	8.80±0.05^C^ ^a^	9.86±0.02^A^	8.97±0.05^B^ ^a^
		gastric	7.99±0.08^B^ ^b^	9.06±0.02^A^	7.39±0.10^C^ ^b^
		enteric I	6.17±0.31^C^ ^c^	8.97±0.03^A^	6.68±0.05^B^ ^a^
		enteric II	6.31±0.10^C^ ^c^	9.01±0.04^A^	6.64±0.02^B^ ^b^
*L. acidophilus* La-5	1	0	8.52±0.05^B^ ^a^	8.86±0.01^A^	7.86±0.03^C^ ^a^
		gastric	5.66±0.22^B^ ^a^	7.86±0.02^A^	4.39±0.02^C^ ^a^
		enteric I	3.71±0.10^C^ ^a^	8.26±0.04^A^	4.90±0.03^B^ ^a^
		enteric II	3.59±0.28^C^ ^a^	8.30±0.01^A^	4.84±0.02^B^ ^a^
	14	0	8.60±0.07^A^ ^a^	8.63±0.08^A^	7.70±0.04^B^ ^b^
		gastric	5.14±0.16^B^ ^b^	7.66±0.02^A^	4.51±0.05^C^ ^a^
		enteric I	3.41±0.10^C^ ^b^	7.77±0.03^A^	4.65±0.03^B^ ^b^
		enteric II	3.66±0.20^C^ ^a^	8.37±0.03^A^	4.62±0.04^B^ ^b^
	28	0	8.26±0.04^B^ ^b^	8.75±0.01^A^	7.65±0.04^C^ ^b^
		gastric	3.91±0.32^B^ ^c^	8.00±0.01^A^	4.49±0.05^B^ ^a^
		enteric I	3.27±0.43^C^ ^b^	8.06±0.01^A^	4.42±0.05^B^ ^c^
		enteric II	3.19±0.34^C^ ^b^	8.03±0.08^A^	4.41±0.04^B^ ^c^
*L. sakei* 2a	1	0	8.09±0.02^C^ ^a^	9.13±0.04^A^	8.24±0.02^B^ ^c^
		gastric	3.00±0.23^C^ ^c^	8.38±0.03^A^	6.17±0.01^B^ ^a^
		enteric I	2.58±0.13^C^ ^b^	8.66±0.02^A^	6.26±0.09^B^ ^a^
		enteric II	2.36±0.14^C^ ^b^	8.74±0.03^A^	6.34±0.05^B^ ^a^
	14	0	8.27±0.03^C^ ^a^	9.26±0.03^A^	8.44±0.01^B^ ^a^
		gastric	4.82±0.32^C^ ^a^	8.55±0.04^A^	6.23±0.02^B^ ^a^
		enteric I	3.36±0.31^C^ ^a^	8.68±0.01^A^	6.35±0.06^B^ ^a^
		enteric II	3.65±0.22^C^ ^a^	8.71±0.02^A^	6.38±0.02^B^ ^a^
	28	0	8.11±0.04^C^ ^a^	9.15±0.01^A^	8.36±0.03^B^ ^b^
		gastric	4.01±0.32^C^ ^b^	8.42±0.05^A^	6.20±0.03^B^ ^a^
		enteric I	2.91±0.33^C^ ^a^	8.41±0.01^A^	6.30±0.02^B^ ^a^
		enteric II	3.11±0.33^C^ ^a^	8.42±0.05^A^	6.31±0.04^B^ ^a^

Populations of *B. animalis* Bb-12, *L. acidophilus* La-5, and *L. sakei* 2a assessed in the probiotic *petit-suisse* cheese (F2) throughout storage (1, 14, and 28 days) at 4°C and during the *in vitro* assay simulating the gastrointestinal conditions [time-point zero, and after 2 h (gastric phase), 4 h (enteric phase I), and 6 h (enteric phase II)]. The samples were analyzed using three methods: plate counts, Real-time PCR (qPCR) and Real-time PCR combined with propidium monoazide (PMA-qPCR).

**Footnote:**

Values are expressed as mean log CFU/g ± standard deviation (SD) obtained by plate count method and as log CFU equivalents/g as calculated from Ct values for qPCR and PMA-qPCR;

A,B,C Different superscript capital letters in a row denote significant differences between methods (*P*<0.05).

a,b,c Different superscript lowercase letters in the same column for each phase denote significant differences between storage days (*P*<0.05).

The three strains studied lost their viability after going through the simulated gastric digestion. However, Bb-12 exhibited a significant better survival capacity than the two *Lactobacillus* strains tested. Bb-12 viable cell loss ranged from less than 0.8 log units in both formulations (day 1) to 1.33 and 1.97 log units, respectively, for F1 and F2 (day 28). In F1, the results of Bb-12 enumeration obtained by plate count were 0.3 log unit lower than those obtained by PMA-qPCR, whereas they were 0.8 log units higher in F2 (*P*<0.05). The decline in La-5 viability was more noticeable, dropping about 2.9 log units for both formulations, as observed on day 1. La-5 viability loss in cheeses F1 and F2 were, respectively, 4.7 and 3.5 log units as shown on day 14 and of 4.1 and 4.4 log units after 28 days of storage, considering the plate count method. On the other hand, with bacterial counts significantly higher, using PMA-qPCR (*P*<0.05), the mean La-5 viability loss was of 3.9 and 3.3 log units for F1 and F2, respectively. The major discrepancy between both methods was observed for *L. sakei* 2a quantification. According to the plate count method, *L. sakei* 2a showed a very poor survival capacity, as shown by its low recovery rates after 2 h under gastric conditions, observed for F1 - 16.0%, 28.0%, and 21.0% and F2 - 36.7%, 59.3%, and 48.5%, respectively on days 1, 14, and 28 (Fig. 1). In contrast, the survival ratio was superior to 74% in all time-points evaluated during the food products storage [except for F1 on day 28 (68.8%)] by the use of PMA-qPCR method, suggesting a survival rate even better than the one assessed for La-5.

During the simulated enteric phases (I and II), the three strains were more tolerant to the enteric conditions than to the previous gastric phase (*P*<0.05) (data not shown). At the end of the enteric phase I, Bb-12 counts had decreased 1 log unit on day 1, reaching a ca. 2 log units reduction on the 28^th^ day of storage, as verified using the plate count method. Using PMA-qPCR technique, Bb-12 populations showed a maximum average reduction of 1 log unit throughout the product shelf-life. On the other hand, at the end of enteric phase II, Bb-12 viability did not change significantly (*P*>0.05) (Tables 3 and 4). The total percentage recovery of Bb-12, relative to the initial levels inoculated into the cheeses, was affected by the storage time, showing a decreasing trend (*P*<0.05) for both formulations regardless the method employed for counting (Figure 1). Considering the PMA-qPCR method, no significant differences on the total percentage recovery for Bb-12 between F1 and F2 were observed, for the three storage periods evaluated, except for day 1, in which recovery was higher in the synbiotic cheese (F1) (*P*<0.05). However, by use of the classic method, F1 exhibited significant lower percentage recovery rates (*P*<0.05).

*L. sakei* 2a showed a higher capacity to resist against the presence of bile and pancreatic juices, when compared to Bb-12, considering the recovery rates obtained (Fig. 1). Similarly to that observed in the gastric phase, higher counts (*P*<0.05) were obtained for *L. sakei* 2a by the use of the PMA-qPCR method, compared to the plate count technique (4.4, 4.3, and 3.9 log units higher for F1 and 3.7, 3.0, and 3.4 log units higher for F2, assessed on days 1, 14, and 28, respectively). In addition, after 2 h of exposure to pH 5.4–5.7 plus bile and pancreatic juices, a recovery effect was observed in F1, relatively to the population of cells surviving the previous gastric phase. Subsequent recovery effects were observed by pH adjustment to 6.8–7.2 during the next two hours (enteric phase II) for both formulations. At the end of the 6 h-assay, *L. sakei* 2a populations were reduced by approximately 62% (plate count) and 25% (PMA-qPCR) of its initial counts. The storage time did not influence the total recovery observed in the F1 (*P*>0.05), whereas a significant decreasing trend was observed in F2 (*P*<0.05).

Similarly, the results obtained for the survival of La-5 were higher (*P*<0.05) when assessed by the use of the molecular method in comparison to the traditional plate count technique. Based on PMA-qPCR, the viability observed for La-5 at the end of the enteric phase I in F1 remained relatively constant (ca. 4.9 CFU/g) throughout the storage of the food product and did not differ from the mean populations verified at the end of enteric phase II (*P*>0.05) (data not shown). On the other hand, at the end of both enteric phases, a decrease in the La-5 viability was observed in F2 during its shelf-life but, although statistically significant (*P*<0.05), they were assessed as less than 0.5 log unit. Differently, the classical method revealed a clear decrease in La-5 viability after enteric phase I (approximately 1.9 log unit at day 1 in both formulations). At the end of the enteric phase II for synbiotic cheese (F1), a small, although statistically significant (*P*<0.05) recovery effect was demonstrated throughout the storage time. This recovery was not observed for F2. The total average reduction in La-5 viability at the end of the *in vitro* assay, assessed in F1 and F2 was respectively 63-59.7% (PMA-qPCR method) and 43.2-41.6% (plate count technique). Overall, considering the three strains studied, Bb-12 showed the highest survival rates, in the range or above 6 log CFU/g, throughout the *in vitro* assay.

### Morphological Changes During the *In Vitro* Assay Simulating the GIT Conditions

**Figure 2:**
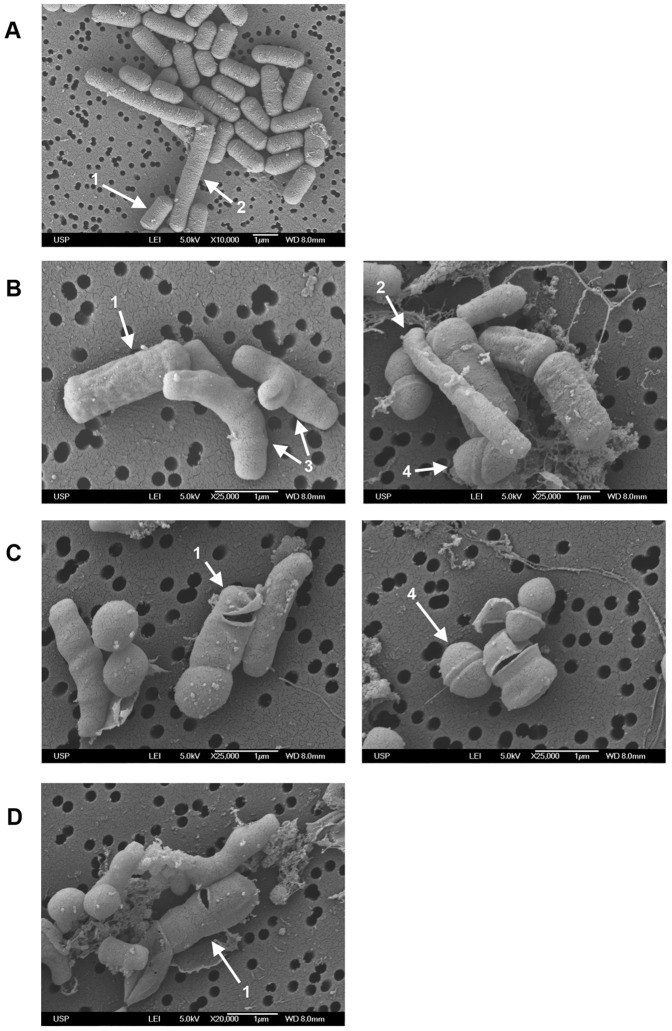
Morphological changes in *S. thermophilus*, La-5, Bb-12, and *L. sakei* 2a during simulated digestive stress. Morphological changes, observed through scanning electron microscopy, in *S. thermophilus*, *L. acidophilus* La-5, *B. animalis* Bb-12, and *L. sakei* 2a throughout the different phases of the in *vitro* assay simulating the gastrointestinal conditions. Over the entire experiment, some representative photographs were obtained at time-point zero (untreated cells) (**A**); after 2 h, gastric phase (pH 2.3–2.6 in the presence of pepsin and lipase, 2 h) (**B**); after 4 h, enteric phase I (pH 5.4–5.7 in the presence of pancreatin and bile, 2 h) (**C**), and after 6 h, enteric phase II (pH 6.8–7.2 in the presence of pancreatin and bile (**D**) are shown. (**1**) *L. sakei* 2a; (**2**) *L. acidophilus* La-5; (**3**) *B. animalis* Bb-12, and (**4**) *S. thermophilus*.

Scanning electron micrographs revealed morphological changes in *S. thermophilus*, *L. acidophilus* La-5, *B. animalis* Bb-12, and *L. sakei* 2a, tested under simulated GIT stress conditions (Fig. 2). Untreated cells exhibited the characteristic rod shape of *Lactobacillus* and a smooth and spherical appearance of *S. thermophilus* (Fig. 2A). However, drastic changes were observed in the cell surface and shape throughout the course of the assay. After 2 h of incubation at pH 2.3–2.6, in the presence of pepsin (3 g/L) and lipase (0.9 mg/L) (gastric phase) the surface of the bacteria clearly seemed to be damaged and the presence of dimples was also observed (Fig. 2B). After exposure to the gastric phase and enteric phase I, the cells demonstrated a shrunken and empty appearance (Fig. 2C). At these incubation times several cells had surface ruptures. Between enteric phases I and II, no further noticeable changes were observed in cells' morphology. At the end of the 6 h-assay, numerous lysed cells and cell debris were verified (Fig. 2D).

## Discussion

Even though inactivated, dead or cellular components of probiotic bacteria are also thought to mediate beneficial effects on the host [37], the current consensus is that probiotics should be alive to exert their beneficial effect(s). In addition to the safety and functionality criteria, the probiotic candidates are chosen for their resistance to passage throughout the GIT and their capacity to transiently colonize the gut. The choice of the food matrix also influences the activity and survival of the probiotic strain until the end of the food product shelf-life and during its passage throughout the GIT [38], [39], [40].

Comparison of the survival data from literature is somehow difficult due to the diversity of types of assays and conditions employed. However, the same protocol and probiotic strains (La-5 and Bb-12) tested in this study were also used by Bedani et al. [41]. The authors showed that fermented soy food matrix improved the survival capacity of the probiotic strains, particularly for Bb-12, when compared to a freshly prepared culture tested under the same stress conditions. The present study (for comparative purposes, only the plate count values are being considered at this point) indicated that *petit-suisse* cheese also improved La-5 and Bb-12 survival against simulated GIT conditions, in comparison with the pure culture values reported by Bedani et al. [41]. However, the survival of La-5 and Bb-12 verified by those authors in the fermented soy matrix was better than the one observed in our *petit-suisse* cheese throughout all the storage period tested.

According to the results obtained by both PMA-qPCR and culture methods, our *petit-suisse* cheeses (F1 and F2) supported the probiotics survival and could be a good alternative as a food matrix to deliver live probiotic microorganisms. The differences in viability loss among Bb-12, La-5, and *L. sakei* 2a strains observed in here further support the idea of the strain-specificity survival capacity [42], [43] and corroborate other studies, suggesting that Bb-12 is one of the most resistant strains to GIT conditions [42].

Contrary to our expectations, the prebiotic mix of inulin and FOS, used in this study, did not show any additional protection for the strains tested in the simulated gastrointestinal environment (Tables 3 and 4). According to the literature, there is some evidence that the introduction of a prebiotic ingredient in a product formulation may improve the viability of probiotic strains during manufacture, storage, and also during *in vitro* conditions of the GIT, even though the underlying mechanisms remain unclear [44].

Possibly, the strains viability would benefit from an assay in which the changes in pH and gastric juice flow are dynamically simulated, as in most advanced digestion systems described in the literature [43], instead of the static model employed in this study. However, the viability rates determined here are within the range of fecal recovery (as intestinal survival indicator) previously reported in *in vivo* interventions with healthy humans [40], [45].

The second question raised in the research here described was to examine the suitability of three different enumeration methods for a reliable determination of bacteria viability in *petit*-*suisse* cheeses during storage and under the stressful conditions of *in vitro* GIT simulation. The results of plate counts, the “gold standard” for viability counts [46], were compared with those obtained by the culture-independent methods, qPCR and PMA-qPCR. Prior studies have shown that the plate counts differed significantly from the results of other viability assays, including qPCR, LIVE/DEAD bacterial viability kit, fluorescent *in situ* hybridization (FISH), and multiparameter flow cytometry [47], [48], [49].

The presence of multiple and phylogenetically highly related strains in our *petit-suisse* cheeses made the differential enumeration of those microorganisms, by the use of plate count technique, a difficult and laborious task due to the similarity in growth requirements and the time of incubation needed to yield the results. Additionally, the differentiation based on morphology of the colonies was necessary to avoid an overestimation of La-5 counts. Similarly, several authors have shown that differential enumeration of bacteria in probiotic foods is often compromised due to the presence of multiple and close related species [30], [50]–[52]. Besides, the distinction based on differences in morphology of the colonies is highly subjective and may lead to an inaccurate estimation of the population of probiotic bacteria in commercial products [30], [53]. Moreover, culture-dependent techniques reflect only the culturable fraction of the bacterial population [19], [20] and it is now known that many Gram-positive and Gram-negative bacteria enter in the so called viable but not culturable state (VBNC) in response to various environmental factors or as a result of sub-lethal injury [54].

In this study, the species-specific primers used allowed an unequivocal detection among the closely related species studied and improved the assay discriminatory power, compared to the use of plate count technique. Another advantage offered by the qPCR method was its speed, since in our work the bacterial quantification was performed in about 4 h, compared to the 72 h required by the use of selective media.

In this study, viable counts obtained by qPCR were slightly higher, but similar to those obtained by PMA-qPCR and plate count at the initial time of the assay, confirming the presence of mainly viable and culturable cells. However, with the intensification of the stress induced throughout the various stages of the assay, the differences between qPCR and the other two methods increased. A decrease in the counts obtained by plate count and PMA-qPCR was observed, while qPCR count values remained fairly similar (Tables 3 and 4). The SEM results confirmed, at the morphological level, the effect of the challenge posed by low-pH and gastric juice for these microorganisms (Fig. 2). The observed decline in viability assessed by plate count and PMA-qPCR methods coincided with the gradual increase in severity of changes in morphology and loss of cell integrity, resulting in the leakage of intracellular contents. Therefore the bacterial populations are overestimated by qPCR, showing the unreliability of the method for the quantification of intact bacteria mixed with large numbers of injured and dead cells. Similar results were observed elsewhere [20], [49], [55] and demonstrated the inability of qPCR method to differentiate between live and dead cells, as its main drawback.

Moreover, the difference among count values obtained by PMA-qPCR and plate methods increased with the intensification of stress during gastric and enteric phases (Tables 3 and 4). The accumulation of injured, VBNC or dead cells unable to form colonies, within the stressed cells, throughout the assay may explain the lower plate count values determined. The existence of probiotic bacteria in the dormant or active, but unculturable state, has been reported before in probiotic products and dairy starters [48], [56]–[59]. On the other hand, in the current study, the PMA treatment efficiently suppressed the amplification of DNA from dead cells, in agreement with Nocker et al. [21], [60], Pan and Breidt [24], and Wuertz and Bae [22]. It may be assumed that part of the sub-lethal injured and VBNC subpopulations maintained its integrity preventing the PMA diffusion. Thus, they could be quantified by the PMA-qPCR method, but not by plate counts technique. In fact, the viability recovery observed by the use of the plate count method, particularly for La-5 and *L. sakei* 2a, after the enteric phases I and II, could be attributed to injured cells needing time and optimal conditions for reparation and recovery. Similar recovery effect was observed for La-5 [27], [41] and for Bb-12 [41], [61], after exposure to the enteric phase of simulated GIT. According to our study, for certain conditions, the use of plate count technique may also not provide a reliable tool to monitor bacterial populations. Therefore, caution must be taken when interpreting/extrapolating viability studies results based on plate counts, as they may not reflex the real physiological bacterial state.

In contrast to the discrepancy observed in our study, a good correlation between PMA-qPCR and the plate count methods was observed elsewhere [23], [62]. A possible explanation for the differences observed among the latter studies and our study might be the drastic conditions used in the present work, which led to an accumulation of injured, VBNC or dead cells, leading to lower population levels detected by plate count technique. Our findings are in line with those published in other studies [48], [49], in which discrepancies in results assessed by plate count and qPCR methods, regarding the application of stress conditions, were observed, whereas in no stress or low-stress conditions a very good correlation between them was obtained.

In conclusion, the *petit-suisse* cheeses supported probiotic survival against *in vitro* simulated gastrointestinal stress conditions and, therefore, are a good alternative food matrixes for the delivery of alive probiotics. Our findings demonstrated that the *in vitro* approach tested, although not an accurate reproduction of the *in vivo* events, allows the standardization of testing conditions and the direct comparison among probiotic strains and different food matrices and ingredients, contributing for the selection of the most appropriate matrix for the delivery of specific probiotic strains. This study has shown that the enumeration method deeply influences the analysis results. Therefore, it should be carefully considered depending on the physiological state of the bacterial population and the main objectives of the study. The plate count method is not a reliable enumeration technique for high-stress conditions, such as those verified in *in vitro* assays, which simulate the gastrointestinal environment. The use of PMA overcomes the inability of the qPCR method to differentiate between alive and dead cells. The combination of PMA and species-specific qPCR in this study allowed a quick and unequivocal way of enumeration of viable closely related species under stress in the probiotic and synbiotic *petit-suisse* cheeses. The bacteriocin production by *L. sakei* 2a in the probiotic (F2) and synbiotic (F1) *petit-suisse* cheeses and its possible role in food safety will be addressed in future studies.
